# The durability of previous examinations for cancer: Danish nationwide cohort study

**DOI:** 10.1080/02813432.2024.2305942

**Published:** 2024-01-22

**Authors:** Jesper Lykkegaard, Jonas Kanstrup Olsen, Sonja Wehberg, Dorte Ejg Jarbøl

**Affiliations:** Research Unit for General Practice, Department of Public Health, University of Southern Denmark, Odense, Denmark

**Keywords:** (MeSH): Early Detection of Cancer, Diagnostic Imaging, Endoscopy, Epidemiology, Reproducibility of results, General practice

## Abstract

**Objective:**

Patients previously examined for cancer with a negative result may present in general practice with ongoing or new symptoms or signs suggestive of cancer. This paper explores the potential existence of a relatively safe period for cancer occurrence after receiving negative examination results for specific types of cancer, including lung (CT thorax), upper gastrointestinal (gastroscopy), colorectal (colonoscopy), bladder (cystoscopy), and breast (clinical mammography).

**Design:**

Register-based time-to-event analyses.

**Setting:**

Denmark.

**Subjects:**

All 3.3 million citizens aged 30–85 years who on January first, 2017, had not previously been diagnosed with the specific type of cancer were categorized based on the time since their most recent examination.

**Main outcome measures:**

Using 1-year follow-up, we calculated the age- and sex-adjusted hazard ratios of being diagnosed with the related cancer, with non-examined individuals as reference. Negative examination results were defined as the absence of a cancer diagnosis within 6 months following the examination.

**Results:**

Previous negative examination results were common, also among those diagnosed with cancer during follow-up. For 10 years after a negative colonoscopy the risk of diagnosing a colorectal cancer was nearly halved. However, already 1 year after a clinical mammography and 2 years after a CT thorax the risk of diagnosing the related cancers was significantly higher among those with a previous negative result compared to non-examined individuals.

**Conclusion:**

This study did not identify a post-examination period in which the cancer risk, compared to non-examined individuals, was sufficiently low to confidently rule out any of the investigated cancers.

## KEY POINTS

What should one expect when considering re-examining a patient with a negative result of a previous examination for cancer? We found that previous negative examination results are common in the general population and among those subsequently diagnosed with cancer. We did not find a safe period after any of the examinations in which a negative result alone could safely rule out the presence of cancer.

## Introduction

Certain examinations, such as computer tomography (CT) of the thorax for lung cancer [1], gastroscopy for upper gastrointestinal cancer [2], colonoscopy for colorectal cancer [3], cystoscopy for bladder cancer [4], and clinical mammography for breast cancer [5] are recognized for their effectiveness in detecting the respective cancer types if present. However, patients who have previously undergone these examinations and received negative results, indicating the absence of cancer, may subsequently present in general practice with ongoing or new symptoms or signs suggestive of a missed or new developed cancer. This poses a challenge for general practitioners (GPs) who must make decisions regarding whether to repeat the examination and, if not, how long to rely on the negative result. Diagnostic considerations include the possibility of a new cancer developing or a previously existing cancer being missed during the initial examination [3,6].

The issue of when to repeat examinations has become increasingly common, particularly in healthcare systems with cancer screening programs and fast track referral systems that lead to a larger proportion of the population undergoing examination. Alarm symptoms associated with cancer are frequently reported, even among individuals without cancer, and it is common for patients who were previously investigated and found to be cancer-free to continue experiencing episodes of symptoms such as cough, dyspepsia, or bleeding [7,8]. Consequently, the question arises: when should the GP again raise the suspicion of cancer?

In this study, we aimed to explore the risk of being diagnosed with cancer during the period following a negative examination result for five commonly performed examinations and their related cancers.

## Methods

### Design and population

**Figure 1.**
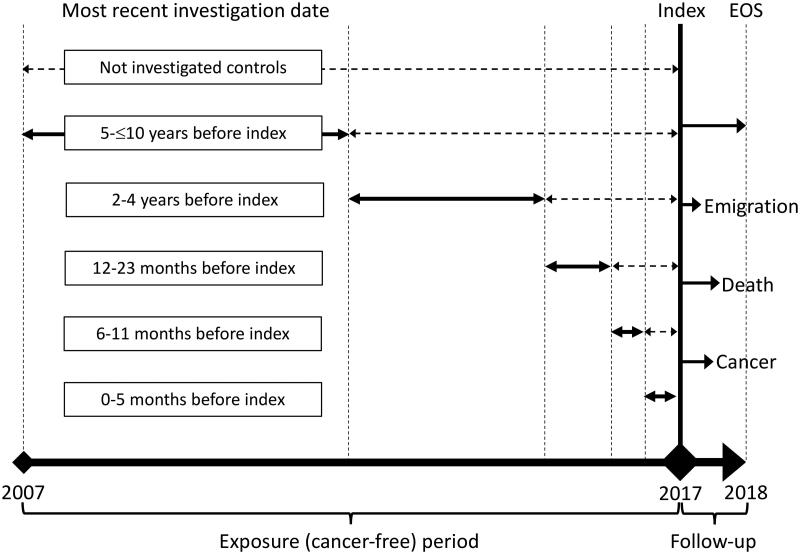
Drawing of the study design. Dashed double arrow lines indicate periods with no investigation. Abbreviation: EOS: End of study.

We conducted a register-based nationwide cohort study, starting on 1 January 2017, (index) to explore five different cancer types (lung, upper gastrointestinal, bladder, colorectal, and breast cancer) and their related examinations (CT thorax, gastroscopy, colonoscopy, cystoscopy, and clinical mammography). The study included all citizens of Denmark aged 30–85 years who had continuous residency in Denmark and no history of the specific cancer within the 10 years before the index date. The lower age limit was determined due to the low incidence of the cancers and examinations below that age, while the upper age limit was set to account for an expected reluctance to examine frail elderly patients increasing their risk of having an undiagnosed cancer. Time-to-event (cancer) analyses were performed, comparing individuals who had undergone examinations during different periods of time before the index date to those who had not been examined (Figure 1). Our design resembles the real-life setting where a patient presents in general practice with or without a previous negative examination result. Like studies on post-colonoscopy colorectal cancer, negative examination results were defined as the absence of a cancer diagnosis within 6 months following the examination [3].

### Setting and data sources

Denmark is a north European country with 5.8 million citizens, each with a unique identification number allowing linkage between national registers. The Danish Civil Registration System contains individual information on age, sex, deaths, and migrations [9]. The Danish National Patient Register (NPR) contains information on all inpatient and outpatient visits to public and private hospital including diagnoses and procedures coded with the International Classification of Diseases 10th revision (ICD-10) [10]. The Danish National Health Service Register collects data from all health contractors in primary care including specialist clinics performing endoscopy [11]. The Danish Cancer Registry contains dates and ICD-10 codes of cancer diagnoses made in the Danish healthcare system [12].

### Cancer diagnostics in Denmark

In Denmark, most healthcare services are fully tax paid including the cancer diagnostic pathways that were introduced in 2008 [13]. The incidence rates of the major cancer types are similar to those in the UK [14]. More than 70% of all patients who are diagnosed with cancer in Denmark started the diagnostic process in general practice. About half of them are diagnosed *via* referral to a cancer diagnostic pathway and the other *via* less linear trajectories [15]. Less than 10% of cancers are diagnosed *via* the national screening programs [16,17]. The Danish national breast cancer screening program, implemented since 2007, offers mammography every second year to women aged 50–69 years. In cases where the mammography leads to suspicion of cancer, a clinical mammography is performed including palpation of breasts and axillary lymph nodes, ultrasound, and fine needle aspiration if necessary [5]. The colorectal cancer screening program, implemented during 2014–2017, involves a test for traces of blood in faeces offered every second year to all citizens aged 50–74 years, followed by a colonoscopy if the result is positive.

### Cancer outcomes and examinations

Table 1 shows the procedure- and diagnosis codes used to identify the examinations and cancers in the registers. We included examinations regardless of their indication, encompassing those performed as part of treatment procedures (e.g. endoscopic polypectomy) and examinations conducted differently from the cancer diagnostic pathways (e.g. including CT-thorax without abdominal scanning and with any radiation dose and contrast use).

**Table 1.** Diagnosis- and procedure codes for cancers and the related investigation.

Cancer type	ICD-10 codes	Investigation	Procedure and service codes
Lung	C33-34	CT-Thorax	UXCC00, UXCC75, or UXCC77
Upper gastrointestinal (Oesophagus or stomach)	C15-16	Gastroscopy	KUJD, KUJD02, or KUJD05 Primary care: 172303, 282303, 086409, 092302, 172307, 282307, 082302, or 082402
Colorectal	C18-C20	Colonoscopy	KUJF32, KUJF35 or KJFA15 Primary care: 082308 or 092114
Breast	C50	Clinical mammography	UXRC40 or UXRC40A
Bladder	C67	Cystoscopy	KUKA, KUKB, KUKB02, KUKB05, KUKC, KUKC02, KUKC05,Primary care: 072306, 092105, 092306, 096404, 096416, 096418, 172304, or 282304

Abbreviations: ICD-10: International classification of diseases 10th revision; primary care are the service codes used. All codes available can be found at Danish Board of Health Data: https://medinfo.dk/sks/brows.php.

### Analyses

We categorized the population based on the time since each individual’s most recent examination before the index date into the following groups: non-examined, 0–5 months, 6–11 months, 12–23 months, 2–4 years, and 5–10 years. It is important to note that in this setup, we conditioned the analysis on survival until the index date. To ensure data anonymity, the groups 6–11 and 12–23 months were merged for upper gastrointestinal and bladder cancer.

From index each individual in the population was followed for 1 year or until a diagnosis of the cancer, death, or migration from Denmark occurred. Using a Fine and Grey competing risk regression, adjusting for age and sex, we calculated the hazard ratio (HR) of being diagnosed with the cancer, using the non-examined group as reference.

As a sensitivity analysis, we repeated the analyses with a follow-up period of only 90 days testing the hypothesis that the associations between cancer risk and previous examination results are stronger in the short run than over a full year. Additionally, considering that cancer growth, doctor-seeking behaviour, test accuracy, and diagnostic decision-making may vary by age and sex, we performed the analyses respectively restricted to older individuals aged 60–85 years and divided by sex.

All analyses were conducted using Stata 17.0 (StataCorp, College Station, TX).

## Results

### Lung cancer and CT thorax

At the index date, the population consisted of a total of 3.3 million individuals aged 30–85 years with no previous lung cancer diagnosis. Among them, 363,114 individuals (11.0%) had undergone a CT thorax in the past 10 years (Table 2). During the 1-year follow-up period, a total of 3696 individuals were diagnosed with lung cancer, out of which 194 individuals (5.2%) had undergone a CT thorax 6–23 months before the index date (Table 3).

**Table 2.** Numbers and proportions of individuals in the Danish 30–85-year-old population, 1 January 2017, who had had a cancer-related examinations during the previous 10 years and had not earlier been diagnosed with the specific type of cancer.

Total population 3,316,784*		Time interval since the last examination before January 1, 2017					
Investigation	Measure	Not exam.	0–5 months	6–11 months	12–23 months	2–4 years	5–10 years
CT Thorax	Number	2,943,747	51,693	44,479	63,768	118,512	84,662
	% row	89.0	1.6	1.3	1.9	3.6	2.6
Clinical mammography	Number	1,411,840	14,883	16,798	30,945	74,578	102,798
	% row	85.5	0.9	1.0	1.9	4.5	6.2
Colonoscopy	Number	2,944,518	36,136	37,711	67,251	120,291	87,072
	% row	89.4	1.1	1.2	2.0	3.7	2.6
Gastroscopy	Number	3,049,257	22,587	21,163	36,101	86,153	99,997
	% row	92.0	0.7	0.6	1.1	2.6	3.0
Cystoscopy	Number	3,160,593	12,636	12,195	20,895	50,495	56,243
	% row	95.4	0.4	0.4	0.6	1.5	1.7

*The total population includes all 30–85 years old persons resident in Denmark on 1 January 2017, and continuously during the 10 years before. For each examination type, the table only includes those not diagnosed with the specific cancer during 10 years before 1 January 2017. For clinical mammography only the female population is included.

**Table 3.** Previous cancer-related examinations among patients in the Danish population who were first-time diagnosed with the cancer during year 2017 and the hazard ratios of being diagnosed with the cancer comparing to the not previously examined persons.

Population 3,316,784*		Time interval since the patient’s latest examination before 1 January 2017					
Cancer/exam.	Measure	Not examined	0–5 months	6–11 months	12–23 months	2–4 years	5–10 years
Lung/CT Thorax	No (% row)	2878 (78)	199 (5.4)	87 (2.4)	107 (2.9)	254 (6.9)	171(4.6)
	HR (CI95)	1 (ref)	2.25 (1.90–2.67)	0.98 (0.74–1.28)	0.94 (0.74–1.19)	1.19 (1.01–1.40)	1.25(1.03–1.51)
Breast/Clinical mammography	No (% row)	3118 (81)	45 (1.2)	41 (1.1)	102 (2.7)	222 (5.7)	346(8.9)
	HR (CI95)	1 (ref)	1.58 (1.17–2.12)	1.26 (0.92–1.71)	1.64 (1.35–2.00)	1.36 (1.18–1.56)	1.37(1.23–1.53)
Colorectal/Colonoscopy	No (% row)	2388 (87)	91 (3.3)	34 (1.2)	54 (2.0)	115 (4.2)	76(2.8)
	HR (CI95)	1 (ref)	1.89 (1.58–2.27)	0.53 (0.38–0.74)	0.57 (0.45–0.73)	0.66 (0.55–0.78)	0.60(0.48–0.74)
Upper gastroint/Gastroscopy	No (% row)	754 (84)	46 (5.1)	32 (3.6)		29 (3.2)	39 (4.3)
	HR (CI95)	1 (ref)	4.90 (3.34–7.18)	1.51 (0.97–2.34)		1.09 (0.71–1.68)	1.21 (0.82–1.78)
Bladder/Cystoscopy	No (% row)	640 (90)	21 (3.0)	11(1.6)		20 (2.8)	16 (2.3)
	HR (CI95)	1 (ref)	2.87 (1.73–4.75)	0.70(0.36–1.36)		0.80 (0.47–1.37)	0.74 (0.43–1.26)

*The total population includes all 30–85 years old persons resident in Denmark on 1 January 2017, and continuously during the 10 years before. For clinical mammography, only the female population is included. Abbreviations: No: total number of patients diagnosed with the cancer type during 2017 among those not diagnosed with it during the previous 10 years; HR(CI95): age- and sex-adjusted one year hazard ratio of being diagnosed with the cancer with 95% confidence interval comparing to non-investigated persons. For upper gastrointestinal- and bladder cancer the 6–11- and 12–23-months groups were collapsed due to low numbers.

**Figure 2.**
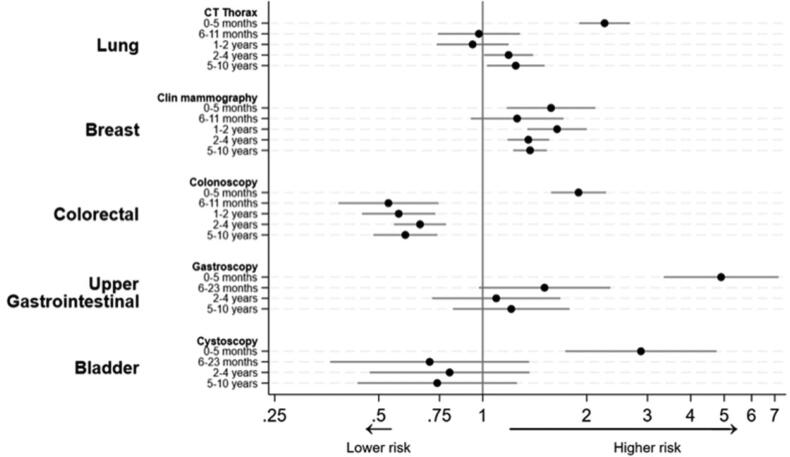
Hazard ratios for cancer after completed examination compared to not-examined persons.

Among those individuals who had a negative CT thorax 6–11 months before the index date, the risk of lung cancer was not significantly different from the risk in the non-examined group, with a HR of 0.98 (95% confidence interval [CI]: 0.74–1.28). However, individuals who had the examination 2–4 years and 5–10 years before the index date had higher risks of lung cancer compared to the non-examined group, with HRs of 1.19 (CI: 1.01–1.40) and 1.25 (CI: 1.03–1.51), respectively (Table 3 and Figure 2).

### Breast cancer and clinical mammography

The female population consisted of a total of 1.7 million individuals aged 30–85 years with no previous breast cancer diagnosis. Among them, 240,002 individuals (14.5%) had undergone a clinical mammography in the past 10 years (Table 2). During follow-up, a total of 3874 individuals were diagnosed with breast cancer, out of which 143 individuals (3.7%) had undergone a clinical mammography 6–23 months before the index date (Table 3).

Compared to the non-examined group, individuals who had a previous negative clinical mammography had higher HRs for breast cancer. The HRs were 1.26 (CI: 0.92–1.71) for those examined 6–11 months before index, 1.64 (CI: 1.35–2.00) for those examined 12–23 months before index, 1.36 (CI: 1.18–1.56) for those examined 2–4 years before index, and 1.37 (CI: 1.23–1.53) for those examined 5–10 years before index (Table 3 and Figure 2).

### Colonoscopy and colorectal cancer

The population consisted of a total of 3.3 million individuals aged 30–85 years with no previous colorectal cancer diagnosis. Among them, 348,461 individuals (10.6%) had undergone a colonoscopy in the past 10 years (Table 2). During the one-year follow-up period, a total of 2758 individuals were diagnosed with colorectal cancer, out of which 88 individuals (3.2%) had undergone a colonoscopy 6–23 months before the index date (Table 3).

Compared to the non-examined group, individuals who had a negative colonoscopy 6–11 months before the index date had a lower HR for colorectal cancer, with an HR of 0.53 (CI: 0.38–0.74). The HRs remained low if the most recent colonoscopy had been performed 1–10 years before the index date (Table 3 and Figure 2).

### Gastroscopy and upper gastrointestinal cancer

The population consisted of a total of 3.3 million individuals aged 30–85 years with no previous upper gastrointestinal cancer diagnosis. Among them, 266,001 individuals (8.0%) had undergone a gastroscopy in the past 10 years (Table 2). During the 1-year follow-up period, a total of 900 individuals were diagnosed with upper gastrointestinal cancer, out of which 32 individuals (3.6%) had undergone a gastroscopy 6–23 months before the index date (Table 3).

Compared to the non-examined group, individuals with a previous negative gastroscopy had a higher HR for being diagnosed with upper gastrointestinal cancer, although this difference was not statistically significant (Table 3 and Figure 2).

### Cystoscopy and bladder cancer

The population consisted of a total of 3.3 million individuals aged 30–85 years with no previous bladder cancer diagnosis. Among them, 152,464 individuals (4.8%) had undergone a cystoscopy in the past 10 years. During the 1-year follow-up period, a total of 708 individuals were diagnosed with bladder cancer, out of which 11 individuals (1.6%) had undergone a cystoscopy 6–23 months before the index date (Table 3).

Compared to the non-examined group, individuals with a previous negative cystoscopy had a lower HR for bladder cancer, although this difference was not statistically significant (Table 3 and Figure 2).

The HRs were similar among persons aged 60–85 years, between the sexes, and when the follow-up was reduced to 90 days (Supplementary tables 1, 2, 3F and 3M).

## Discussion

### Principal findings

We found that there was no specific period of time after any of the five examinations in which a negative result could definitively rule out the presence of the related cancer. The duration of the negative results’ effectiveness varied among the different examinations, with colonoscopy showing the highest durability and clinical mammography showing the lowest. Even within 1 year after a clinical mammography and 2 years after a CT thorax, the risk of being diagnosed with the related cancer was significantly higher among individuals who had previously received a negative result compared to those who were not examined. A significant proportion of the general population, who were initially cancer-free, had previously undergone these examinations with negative results, including individuals who were later diagnosed with the related cancer.

### Strengths and limitations

The registers utilized in our study have high levels of completeness and validity, primarily due to their close integration with payment systems and electronic medical records within the healthcare system [12]. The examinations included in our study are predominantly performed in public and private hospitals, as well as specialist clinics, all of which report their data to the registers.

It is important to note that the registers only record the dates of examinations and diagnoses, without providing specific details about the results of the examinations. However, it is expected that all identified cancer cases would be appropriately recorded in the Cancer Registry. A validation study focusing on colon cancer data revealed that, out of 11,747 cases, only 66 had a difference in diagnosis dates exceeding 180 days, with the Cancer Registry mostly containing the earliest recorded date [18]. This finding supports our assumption that examinations were negative if there was no record of cancer after 6 months.

It deserves mentioning that it is good practice to label some types of examination findings as *probably benign* and consequently order a repeat examination e.g. after 6 months reducing the diagnostic challenge for the GP and the patient. However, such examinations most likely only comprise a minority in this study.

We included all indications for the examinations included in our study. Some were performed for other reasons than cancer suspicion and some of the included coloscopies and clinical mammographies were performed as part of the national screening programs. All the included examinations should detect the related type of cancer if present, also if conducted for other reasons than cancer suspicion. The indication for an examination is likely to be closely linked to the patient’s individual cancer risk profile, thereby affecting the durability of a negative examination result.

The presence of a previous negative examination result may affect the patients’ healthcare seeking behavior and the GPs’ preferences for examining. Both would affect the chances of diagnosing present cancers potentially biasing our comparisons of cancer HRs between examined and non-examined persons.

We did not have access to data regarding the specific subtypes of the examinations or other sensitivity-increasing diagnostic procedures that patients may have undergone, such as bronchoscopy, urine cytology, or additional imaging. In the clinical setting, this information is relevant but, similar to our study, not always available or reliably reported by patients.

### Comparison with existing literature

Our findings have important implications for GPs, patients, and hospitals when considering the timing of repeat examinations following a negative result. Strictly relying on a ‘safe period’ after a negative examination result is not justified. Instead, all available information should be considered, including patient risk factors, signs and symptoms, findings from supplementary examinations, risk-increasing findings from the negative examination (such as nodules and polyps), and knowledge about the examiner’s skills [19,20].

To understand why a recent negative examination result does not guarantee the absence of cancer, we need to consider three influencing factors: the sensitivity of the examination and examiner at the time of the test, the growth rate of the cancer, and the differential risk factors and symptomatology between examined and non-examined individuals [3].

While the sensitivity of these examinations is generally high, meaning the risk of overlooking a significant cancer is low, it is important to note that post-examination cancers can still occur [3–5,21]. Cancers can develop rapidly within months, which can occur shortly after a negative examination and be difficult to distinguish from a false negative result.

The key to understanding why the risk of cancer can be higher in examined individuals compared to non-examined individuals lies in the fact that all examinations are performed based on clinical indications or positive findings in screening (such as high mammographic density or occult blood in feces) [22]. Some cancer risk factors and contributing causes may persist in patients with negative examination results, including hereditary factors, lifestyle and other exposures, chronic symptoms, clinical signs, and healthcare-seeking behavior.

Our study adds to existing research demonstrating that relying too long on negative findings can lead to delays in cancer diagnosis [23,24]. The ability to detect and remove cancer and precursors during endoscopy may explain why the HRs for colonoscopy and cystoscopy are lower compared to other examinations (Figure 2) [2,4,19]. Our findings can be generalized to healthcare systems similar to the UK, with comparable cancer incidence rates to Denmark, publicly funded fast-track cancer examinations, and national screening programs for breast and colorectal cancer [14,25,26]. The rates of post-colonoscopy and post-gastroscopy cancers observed in our study align with recent findings from the US and UK [3,21,27,28].

### Implications for research and/or practice

Our findings have important implications for both research and clinical practice. Firstly, it is evident that a negative colonoscopy provides a greater duration of reduced risk compared to a negative CT-thorax or clinical mammography. However, even in the case of colonoscopy, the risk reduction demonstrated does not render the risk of cancer negligible. Therefore, patients, GPs, and those involved in triaging GP referrals should exercise great caution when considering the use of a recent negative examination result to rule out cancer.

It is crucial to acknowledge that the results of the included examinations rarely provide a definitive ‘yes’ or ‘no’ answer regarding the presence of cancer. Certain examination findings, such as a small nodule on a CT-thorax considered benign, removed polyps during endoscopy, or the need for a fine needle aspiration as part of a clinical mammography, are associated with a higher subsequent risk of cancer. Unfortunately, our study did not include this information, along with the indication for performing the examination and patient-related risk factors, symptoms, and signs. It is important for GPs to collect and balance these factors when considering the need for repeat examinations. Future research should explore how these additional factors influence the estimates of risk.

The rates of post-examination cancers are expected to improve over time as the accuracy of the examinations increases [3,21]. The durability of negative examination results will also increase as the a priori risk of the examined population decreases, which happens with increased screening and availability of the examinations. However, it is unlikely that previous negative results from the current types of examinations included in our study will ever completely rule out the occurrence of cancer, even in the short term.

## Supplementary Material

Supplemental Material

Supplemental Material

Supplemental Material

Supplemental Material

## Data Availability

Data supporting the findings of this study was used under a license granted specifically for the current study and therefore is not publicly available according to the data protection regulations of Danish Data Protection Agency, Statistics Denmark and the Danish Health and Medicines Authority.
