# Supplementary material for: The durability of previous examinations for cancer: Danish nationwide cohort study
Source: Scand J Prim Health Care. 2024 Jan 22;42(2):246–53. doi: 10.1080/02813432.2024.2305942 (PMC11003324; doi:10.1080/02813432.2024.2305942)
Supplement: Supplemental Material [file IPRI_A_2305942_SM2642.docx]

| **Supplementary table 3M: Previous cancer-related examinations among patients in the Danish population who were first-time diagnosed with the cancer during year 2017 and the hazard ratios of being diagnosed with the cancer comparing to the not previously examined persons – MALES ONLY** | | | | | | | |
| --- | --- | --- | --- | --- | --- | --- | --- |
| Population 1,628,119* | | Patients diagnosed with the cancer and the time interval since their last examination | | | | | |
| Cancer/exam. | Measure | Not exam | 0-5 months | 6-11 months | 12-23 months | 2-4 years | 5-10 years |
| Lung/  CT Thorax | No (% row) | 1409(77.4) | 103 (5.7) | 41 (2.3) | 62 (3.4) | 119 (6.5) | 87 (4.8) |
|  | HR(CI95) | 1 (ref) | 2.11 (1.66-2.68) | 0.88 (0.60-1.31) | 0.94 (0.67-1.32) | 1.15 (0.91-1.46) | 1.15 (0.86-1.53) |
| Breast/Clinical  mammography | No (% row) | 28 (100) | 0 (0.0) | 0 (0.0) | 0 (0.0) | 0 (0.0) | 0 (0.0) |
|  | HR(CI95) | 1 (ref) | 0.00 (.-.) | 0.00 (.-.) | 0.00 (.-.) | 0.00 (.-.) | 0.00 (.-.) |
| Colorectal/  Colonoscopy | No (% row) | 1274 (87.6) | 49 (3.4) | 18 (1.2) | 21 (1.4) | 59 (4.1) | 34 (2.3) |
|  | HR(CI95) | 1 (ref) | 1.80 (1.40-2.29) | 0.47 (0.30-0.75) | 0.45 (0.32-0.65) | 0.66 (0.52-0.84) | 0.52 (0.38-0.71) |
| Upper gastroint/  Gastroscopy | No (% row) | 525 (84.1) | 33 (5.3) | 19 (3.0) | | 18 (2.9) | 29 (4.7) |
|  | HR(CI95) | 1 (ref) | 4.58 (2.85-7.37) | 1.18 (0.65-2.15) | | 1.21 (0.73-1.99) | 1.45 (0.93-2.25) |
| Bladder/  Cystoscopy | No (% row) | 469 (89.7) | 14 (2.7) | 9 (1.7) | | 17 (3.3) | 14 (2.7) |
|  | HR(CI95) | 1 (ref) | 2.38 (1.33-4.24) | 0.62 (0.29-1.32) | | 0.74 (0.41-1.35) | 0.75 (0.42-1.34) |
| *The total population includes all 30-85 years old male residents in Denmark on January 1^st^, 2017, and continuously during the ten years before. Abbreviations: No, total number of persons diagnosed with the cancer type during 2017 among those not diagnosed with it during the previous ten years; HR(CI95), age-adjusted one year hazard ratio with 95% confidence interval compared to non-investigated persons. For upper gastrointestinal- and bladder cancer the 6-11- and 12-23-months groups were collapsed due to low numbers. | | | | | | | |
