# Supplementary material for: The durability of previous examinations for cancer: Danish nationwide cohort study
Source: Scand J Prim Health Care. 2024 Jan 22;42(2):246–53. doi: 10.1080/02813432.2024.2305942 (PMC11003324; doi:10.1080/02813432.2024.2305942)
Supplement: Supplemental Material [file IPRI_A_2305942_SM2551.docx]

| **Supplementary table 3F: Previous cancer-related examinations among patients in the Danish population who were first-time diagnosed with the cancer during year 2017 and the hazard ratios of being diagnosed with the cancer comparing to the not previously examined persons – FEMALES ONLY** | | | | | | | |
| --- | --- | --- | --- | --- | --- | --- | --- |
| Population 1,688,665* | | Patients diagnosed with the cancer and the time interval since their last examination | | | | | |
| Cancer/exam. | Measure | Not exam | 0-5 months | 6-11 months | 12-23 months | 2-4 years | 5-10 years |
| Lung/  CT Thorax | No (% row) | 1469 (78.4) | 96 (5.1) | 46 (2.5) | 45 (2.4) | 135 (7.2) | 84 (4.5) |
|  | HR(CI95) | 1 (ref) | 2.40 (1.89-3.05) | 1.07 (0.73-1.57) | 0.93 (0.66-1.30) | 1.22 (0.97-1.52) | 1.33 (1.03-1.73) |
| Breast/Clinical  mammography | No (% row) | 3118 (80.5) | 45 (1.2) | 41 (1.1) | 102 (2.6) | 222 (5.7) | 346 (8.9) |
|  | HR(CI95) | 1 (ref) | 1.58 (1.17-2.12) | 1.26 (0.92-1.71) | 1.64 (1.35-2.00) | 1.36 (1.18-1.56) | 1.37 (1.23-1.53) |
| Colorectal/  Colonoscopy | No (% row) | 1114 (85.5) | 42 (3.2) | 16 (1.2) | 33 (2.5) | 56 (4.3) | 42 (3.2) |
|  | HR(CI95) | 1 (ref) | 2.02 (1.53-2.66) | 0.61 (0.38-0.99) | 0.73 (0.52-1.01) | 0.65 (0.50-0.84) | 0.69 (0.51-0.92) |
| Upper gastroint/  Gastroscopy | No (% row) | 229 (83.0) | 13 (4.7) | 13 (4.7) | | 11 (4.0) | 10 (3.6) |
|  | HR(CI95) | 1 (ref) | 5.57 (2.93-10.59) | 2.18 (1.15-4.15) | | 0.87 (0.39-1.98) | 0.77 (0.34-1.74) |
| Bladder/  Cystoscopy | No (% row) | 171 (92.4) | 7 (3.8) | 7 (3.8) | | | |
|  | HR(CI95) | 1 (ref) | 6.55 (2.42-17.78) | 0.92 (0.43-1.96) | | | |
| *The total population includes all 30-85 years old female residents in Denmark on January 1^st^, 2017, and continuously during the ten years before. Abbreviations: No, total number of persons diagnosed with the cancer type during 2017 among those not diagnosed with it during the previous ten years; HR(CI95), age-adjusted one year hazard ratio with 95% confidence interval compared to non-investigated persons. For upper gastrointestinal cancer the 6-11- and 12-23-months groups and for bladder cancer the 6-11-, 12-23 months, 2-4-, and 5-10 years groups were collapsed due to low numbers. | | | | | | | |
