# Supplementary material for: The durability of previous examinations for cancer: Danish nationwide cohort study
Source: Scand J Prim Health Care. 2024 Jan 22;42(2):246–53. doi: 10.1080/02813432.2024.2305942 (PMC11003324; doi:10.1080/02813432.2024.2305942)
Supplement: Supplemental Material [file IPRI_A_2305942_SM2550.docx]

| **Supplementary table 2: Previous cancer-related examinations among patients in the Danish population who were first-time diagnosed with the cancer during the first 90 days of 2017 and the hazard ratios of being diagnosed with the cancer related to whether and when the examination was completed** | | | | | | | |
| --- | --- | --- | --- | --- | --- | --- | --- |
| Population 3,316,784* | | Patients diagnosed with the cancer and the time interval since their last examination | | | | | |
| Cancer/exam. | Measure | Not exam | 0-5 months | 6-11 months | 12-23 months | 2-4 years | 5-10 years |
| Lung/  CT Thorax | No (% row) | 702 (73.97) | 92 (9.69) | 20 (2.11) | 32 (3.37) | 56 (5.90) | 47 (4.95) |
|  | HR(CI95) | 1 (ref) | 4.67 (3.48-6.25) | 1.03 (0.55-1.93) | 1.34 (0.83-2.16) | 1.13 (0.76-1.68) | 1.55 (1.03-2.32) |
| Breast/Clinical  mammography | No (% row) | 816 (79.45) | 14 (1.36) | 13 (1.27) | 20 (1.95) | 69 (6.72) | 95 (9.25) |
|  | HR(CI95) | 1 (ref) | 1.88 (1.10-3.19) | 1.55 (0.90-2.68) | 1.17 (0.74-1.85) | 1.58 (1.23-2.03) | 1.46 (1.18-1.81) |
| Colorectal/  Colonoscopy | No (% row) | 584 (79.67) | 72 (9.82) | 10 (1.36) | 14 (1.91) | 33 (4.50) | 20 (2.73) |
|  | HR(CI95) | 1 (ref) | 5.91 (4.73-7.38) | 0.50 (0.25-1.00) | 0.55 (0.33-0.90) | 0.68 (0.48-0.97) | 0.66 (0.44-1.00) |
| Upper gastroint/  Gastroscopy | No (% row) | 172 (74.14) | 34 (14.66) | 0 (0.00) | | 10 (4.31) | 6 (2.59) |
|  | HR(CI95) | 1 (ref) | 19.62 (11.95-32.21) | 2.52 (1.10-5.77) | | 1.46 (0.59-3.59) | 1.57 (0.69-3.58) |
| Bladder/  Cystoscopy | No (% row) | 166 (83.00) | 18 (9.00) | 16 (8.00) | | | |
|  | HR(CI95) | 1 (ref) | 10.09 (5.67-17.98) | 0.98 (0.54-1.78) | | | |
| *The total population includes all 30-85 years old persons resident in Denmark on January 1^st^, 2017, and continuously during the ten years before. For clinical mammography, only the female population is included. Abbreviations: No, total number of persons diagnosed with the cancer type during 2017 among those not diagnosed with it during the previous ten years; HR(CI95), age- and sex-adjusted one year hazard ratio with 95% confidence interval compared to non-investigated persons. For upper gastrointestinal cancer the 6-11- and 12-23-months groups were collapsed due to low numbers. For bladder cancer the 6-11 month, 12-23 month, 2-4 years, and 5-10 years were collapsed due to low numbers. | | | | | | | |
