# Supplementary material for: The durability of previous examinations for cancer: Danish nationwide cohort study
Source: Scand J Prim Health Care. 2024 Jan 22;42(2):246–53. doi: 10.1080/02813432.2024.2305942 (PMC11003324; doi:10.1080/02813432.2024.2305942)
Supplement: Supplemental Material [file IPRI_A_2305942_SM2535.docx]

| **Supplementary table 1: Previous cancer-related examinations among patients ≥60 years of age in the Danish population who were first-time diagnosed with the cancer during year 2017 and the hazard ratios of being diagnosed with the cancer related to whether and when the examination was completed** | | | | | | | |
| --- | --- | --- | --- | --- | --- | --- | --- |
| Population 1,295,604* | | Patients diagnosed with the cancer and the time interval since their last examination | | | | | |
| Cancer/exam. | Measure | Not exam | 0-5 months | 6-11 months | 12-23 months | 2-4 years | 5-10 years |
| Lung/  CT Thorax | No (% row) | 2385 (76.59) | 175 (5.62) | 83 (2.67) | 92 (2.95) | 227 (7.29) | 152 (4.88) |
|  | HR(CI95) | 1 (ref) | 2.13 (1.78-2.54) | 1.03 (0.78-1.36) | 0.84 (0.65-1.10) | 1.15 (0.97-1.37) | 1.19 (0.96-1.46) |
| Breast/Clinical  mammography | No (% row) | 1907 (83.53) | 21 (0.92) | 15 (0.66) | 45 (1.97) | 89 (3.90) | 206 (9.02) |
|  | HR(CI95) | 1 (ref) | 1.73 (1.13-2.66) | 1.07 (0.65-1.79) | 1.66 (1.23-2.24) | 1.16 (0.93-1.44) | 1.40 (1.22-1.62) |
| Colorectal/  Colonoscopy | No (% row) | 1988 (86.14) | 75 (3.25) | 30 (1.30) | 47 (2.04) | 99 (4.29) | 69 (2.99) |
|  | HR(CI95) | 1 (ref) | 1.75 (1.43-2.13) | 0.53 (0.37-0.75) | 0.52 (0.40-0.69) | 0.60 (0.50-0.73) | 0.59 (0.47-0.74) |
| Upper gastroint/  Gastroscopy | No (% row) | 588 (82.35) | 41 (5.74) | 28 (3.92) | | 22 (3.08) | 35 (4.90) |
|  | HR(CI95) | 1 (ref) | 5.05 (3.37-7.59) | 1.51 (0.94-2.43) | | 1.00 (0.61-1.62) | 1.28 (0.85-1.94) |
| Bladder/  Cystoscopy | No (% row) | 551 (90.63) | 15 (2.47) | 9 (1.48) | | 17 (2.80) | 16 (2.63) |
|  | HR(CI95) | 1 (ref) | 2.02 (1.11-3.68) | 0.57 (0.27-1.20) | | 0.72 (0.41-1.28) | 0.78 (0.46-1.32) |
| *The total population includes all 60-85 years old persons resident in Denmark on January 1^st^, 2017, and continuously during the ten years before. For clinical mammography, only the female population is included. Abbreviations: No, total number of persons diagnosed with the cancer type during 2017 among those not diagnosed with it during the previous ten years; HR(CI95), age- and sex-adjusted one year hazard ratio with 95% confidence interval compared to non-investigated persons. For upper gastrointestinal- and bladder cancer the 6-11- and 12-23-months groups were collapsed due to low numbers. | | | | | | | |
